# Supplementary material for: Quality of hypertension management in public primary care clinics in Malaysia: An update
Source: PLoS One. 2020 Aug 11;15(8):e0237083. doi: 10.1371/journal.pone.0237083 (PMC7418969; doi:10.1371/journal.pone.0237083)
Supplement: Attachment — Submitted filename: review on PLOS ONE manuscript.docx [file pone.0237083.s001.docx]

The present study aimed to assess management, and the association between the care process and the blood pressure control of hypertensive patients in primary care clinics in Malaysia.

Introduction

The authors were able to address the main issues addressed in the study, demonstrating how the number of hypertensive patients worldwide is growing, and the control of blood pressure in countries still leaves much to be desired. However, some changes would be interesting:

1- Add an excerpt contextualizing what primary care is, for a better understanding of the work even by people who do not have knowledge in this area specifically, something important in the case of work with great informative relevance.

2- Define a more specific objective of the study, and add the authors' hypotheses, what did you expect as a result before producing this study?

Methods

The experiments and evaluations chosen were selected for the purpose of study, providing the necessary data to reach a consolidated conclusion.

Only one suggestion would be to develop this section further:

1 - If possible, the authors will have access to this information, cite the methods of analysis and equipment that were used, in the analysis of the lipid profile, for example.

Results

With the data provided in this section it is possible to easily answer the questions of the present study. Tables and figures in general are very explanatory, observing only these, it is possible to understand the main results, without the need to read the entire body of the text.

However, there are still some suggestions and changes that must be rethought:

1- Changing the order of the tables, I believe that this would facilitate understanding, as an example:

• Table 1: Patient demographic data

• Table 2: Process of care for hypertensive patients

• Table 3: Blood pressure control status in different subgroups of hypertensive patients

• Table 4: Prescribing pattern of antihypertensive agents

2- Add the data from the excerpt “Unfortunately, less than one percent of the records documented assessment of cardiovascular risk” (page 10, line 164), in the body of the text, but not in the table.

3- How was the definition of appropriate choice or not of antihypertensive agents? It is an important aspect that the text does not make clear.

4- Were patients who were undergoing lifestyle changes likewise prescribed hypertensive agents? Explain more about it in the text, it doesn't seem to be clear.

5- Thinking about improving the understanding of the characteristics of the patients analyzed in this research, it would be interesting to include a table with blood pressure data, in addition to only “controlled” and “uncontrolled”. For example:

- Patients Age> 80 years (n = 517)
  - X patients BP systolic 140-150 mmHg and BP diastolic 80-89.
  - Y patients BP systolic 150-160 mmHg and BP diastolic 90-99.

Discussion

In general, it addresses the main subjects of the present work, updating and agreeing with previously published works, showing that the primary care care processes have been improved, concluding on the general question of the study.

Just a few suggestions:

1- In the excerpt “Surprisingly, in this study, in the association between counseling on salt intake and good BP control was observed” (page 20, line 298), was this response expected? What are the possible reasons for this response?

2- According to the results, the authors indicate that patients who had counseling on physical exercise are more likely to have greater control of blood pressure, how was this counseling performed?

3- Add possible study limitations.
