# Supplementary material for: Quality of hypertension management in public primary care clinics in Malaysia: An update
Source: PLoS One. 2020 Aug 11;15(8):e0237083. doi: 10.1371/journal.pone.0237083 (PMC7418969; doi:10.1371/journal.pone.0237083)
Supplement: Attachment — Submitted filename: Response to Reviewers.docx [file pone.0237083.s002.docx]

Dear Editor and Reviewers,

We thank you for the detailed review and constructive comments. The introduction, method, results and discussion sections have been further elaborated. Please find our response to your comments as listed below in the tables.

**Responses to comments from Reviewer #1**

| **Comments** | **Author’s Response** |
| --- | --- |
| **INTRODUCTION** | |
| 1. The authors provide general information about BP management worldwide and in developing countries, but the central problem remained unclear. What is the central point to be demonstrated and why? In short, the problem and purpose of the work should be clearly defined. | Thank you for pointing this out.  We have added a few points and rephrased the section (line 80-89) in the introduction section and hope it would provide more clarity on the problem and purpose of this study.  It now reads:  **Introduction, line 80-89, page 5-6**  “Although there were some evaluations on hypertension, process of care evaluation was last done 10 years ago [11]. Most of the evaluations on hypertensive care focused on the burden of hypertension in the Malaysia population [14–16]. Similarly, evaluations on process of care and BP control in low- and middle-income countries are scarce.  We hypothesized that hypertensive patients who received care for hypertension were associated with better BP control. We also determined the extent of adherence to national clinical practice guidelines (CPG) for hypertension. Therefore, we aimed to evaluate the current process of care for hypertension management as well as to explore the association between process of care and BP control among hypertensive patients in public primary care clinics in Malaysia.” |
| **METHODS** | |
| 1. It seems that the only measures of BP management were "types of counseling provided as well as the prescribed antihypertensive medications". How the types of counseling have been effectively assessed and what about the quantity and quality of these interventions? As for the medications, what information has been extracted (type, dose etc). Please detail these aspects. | We have added some sentences to improve clarity in both measure and statistical analysis sub-sections in the method section:  **Method, line 120-133, page 7-8**  “The process of care included documentation of patients’ physical examinations such as systolic BP and diastolic BP, weight, height, waist circumference, body mass index (BMI), electrocardiography test and fundus examinations. Laboratory investigations including blood glucose, serum creatinine, fasting lipid profile, and urinalysis for micro albuminuria were extracted. All physical examinations and laboratory investigations done within the past one year were considered with the exception of weight and BMI measured within past six months as well as BP assessed at the current visit for better accuracy. In addition, process of care on CV risk assessment such as Framingham Risk Score performed in the past one year and types of counseling provided were recorded. Types of counseling extracted included counseling on diet education, exercise/physical activity, smoking cessation, salt intake and alcohol intake. Data on prescription were also extracted which included the types and number of antihypertensive agents. The antihypertensive agents prescribed were categorized into i) one drug, ii) two drugs and iii) ≥ three drugs. Those without anti-hypertensive agents prescribed but with a diagnosis of hypertension were considered to be on lifestyle modification.”  and  **Method, line 154-163, page 8-9**  “Multivariable logistic regression using complex survey design to account for clustering effect within clinics was used to estimate the adjusted odds ratio and 95% confidence interval for the association of process of care and prescribing practice on BP control. Complete case analysis was performed for the regression. The process of care variables included in the regression were documentation of BMI, electrocardiography, funduscopy, fasting/random blood glucose, creatinine, LDL-cholesterol, urine albumin, Framingham Risk Score, smoking cessation counseling, diet education counseling, salt intake counseling, alcohol intake counseling, and exercise/physical activity counselling. The variable used for prescribing practice was number of antihypertensive agents prescribed.”  We are unable to provide the information on how effective the counseling was being assessed as only type of counseling being provided were documented in the medical record. Similarly, only types of antihypertensive drugs that was prescribed to the patients were extracted in the EnPHC-EVA: Facility study. Hence, we are unable to quantify the dose and duration too.  We have added these limitations in the discussion section:  **Discussion, line 351-358, page 26**  “Third, the information on counseling was limited to types of counseling documented in the medical records. It would be difficult to capture the effectiveness of counseling given to the patients during the doctor-patient consultation session. Thus, we assumed that counseling was delivered accordingly by the attending primary care doctors. Similarly, information on the dose and duration of the antihypertensive agents were not extracted because it was assumed that the dose titration process will occur over time, aiming at BP control. Hence, it is not feasible to measure antihypertensive medication titration on BP control with our cross-sectional study design.” |
| 2. What independent comparisons have been performed through the t-tests and Mann-Whitney tests? What were the processes of care and prescribing patterns inserted in the multivariate analysis? | We have omitted the sentence “Independent t-test /Mann-Whitney U test for continuous variables and chi square test for categorical variables which were used as comparison where applicable” in the statistical analysis section since we did not carry out this analysis.  We have added some sentences in the statistical analysis subsection in the method section (**Method, line 120-133, page 7-8)** to improve the clarity on the variables for process of care and prescribing practice that were used in the multivariable analysis. The change was discussed above in our response to comment #1 in Methods. |
| 3. "Complete case analysis was performed for the regression" - did the authors considered the use of intention to treat analysis? Information about the drop outs within the more than 13,000 cases would be useful. How many patients did not comply to the prescribed treatment or failed to perform reasssessment during the study? | Thank you for the suggestion. However, we are unable to use intention to treat analysis because our study is cross-sectional in nature. Also, we are unable to provide any information on patient compliance to prescribed treatment and dropouts as patients were not followed up as this is not a cohort study.  We have included a sentence on the dropouts and compliance in the statistical analysis sub-section in the method section to improve clarity.  **Method, line 165-166, page 9**  “This was a cross sectional study on hypertension management, thus we could not include dropouts and compliance as our covariates.” |
| **RESULTS** | |
| 1. Overall, although interesting, data seem to reproduce information extensively acknowledged in the literature. This is an important issue to be commented by the authors. Moreover, some aspects must be further clarified. For instance, how "Inappropriate choice of antihypertensive agents" was defined? | Thank you for pointing this out. We agree with the reviewer that the hypertensive management findings were similar with several previous studies. These similar findings showed that the existing hypertensive care in Malaysia remains much as it was in the past decade.  We are adding a heading for conclusion section to improve the structure of the paper. We have incorporated the point and rephrased in the conclusion section. It now reads:  **Conclusion, line 365-375, page 27**  “**Conclusion**  Our present findings show that BP control was suboptimal in public primary care clinics. The process of care remains deficient with gaps in guidelines and actual clinical practice. These suboptimal BP control and hypertensive management findings were consistent with several local cross-sectional surveys and clinical audits on hypertension management conducted in primary care [7,9,11,17,21,22,28,30]. These consistent findings as previous studies demonstrated that the hypertensive care in Malaysia still remains much as it was in the past decade. Therefore, a continuous and concerted efforts are warranted to improve the quality of hypertensive management. Apart from looking at traditional issues of physician awareness of hypertension and physician adherence to guidelines, an evaluation on the how the process being undertaken is important in order for us to understand the dynamics within a primary care clinic.”  Besides that., we have defined the "Inappropriate choice of antihypertensive agents" in the method section to improve clarity.  **Method, line 142-150, page 8**  “Those antihypertensive drugs that were prescribed not according to criteria set in the Malaysian CPG on Hypertension 2018 were considered as inappropriate choice of antihypertensive agents [18]. These drugs were considered inappropriate choice if any of the following was fulfilled: i) alpha-blocker was prescribed as a single agent to hypertensive patients with T2DM or elderly patients ≥ 65 years old ii) beta-blocker was prescribed as a single agent to hypertensive patients with T2DM or dyslipidemia, iii) diuretic was prescribed as a single agent for hypertensive patients with T2DM or dyslipidemia, iv) angiotensin converting enzyme inhibitor (ACEI) or angiotensin receptor blocker (ARB) was not prescribed to T2DM patients with proteinuria.” |
| 2. Only 2.5% of patients received counseling in regards to lifestyle modifications, which seems to be a very small proportion to allow statements about the effectiveness of these procedures to improve BP control. Moreover, information about the characteristics of patients that received this type of counseling would be useful to a better understanding of these findings. | Thank you for pointing this out. We have defined hypertensive patient in the lifestyle modification group and incorporated this definition in the method section to make it clearer:  **Method, line 132-133, page 8**  “Those without anti-hypertensive agents prescribed but with a diagnosis of hypertension were considered to be on lifestyle modification.”  We have added information about BP status of these two groups (HPT with antihypertensive agents and HPT on lifestyle modification) in the result section, both in text (line 196-197) and in Table 3, to further improve clarity:  **Result, line 196-197, page 15**  “Hypertensive patients with lifestyle modification appeared to have a better BP control rate compared to those on antihypertensive agents.”  As for Table 3, we have inserted the two parameters (HPT with antihypertensive agents and HPT on lifestyle modification) with results.  We also changed one of the column headers in Table 3 [from Patients achieving target blood pressure control to n(%)] due to overlook from our side. Kindly refer to Table 3 at :  **Result, line 199-200, page 16-17**  We also have rephrased the discussion section to add further clarity. It now reads:  **Discussion, line 312-317, page 24**  “Our findings indicate that hypertensive patients who had documented counseling on exercise/physical activity had 1.3 odds of having their BP controlled compared to those not receiving counseling. However, the information on how counseling was performed was not available in the medical record. We assume that the counseling was done as the usual practice in the clinic. The association of exercise/physical activity with lower BP was also reported in a previous study [37].” |
| 3. Finally, please explain why to take two or more antihypertensive agents was inversely associated with BP management – is this due to the treatment or to the fact that those patients had higher BP levels. The way the text is written may lead to misinterpretation of these findings. | Thank you for pointing this out.  We too were surprised with this finding which was not in line with the literature where patients who were prescribed with combination therapy appeared to have better BP control than those who were on monotherapy alone.  To avoid misinterpretation, we have added a sentence in the discussion section about the unexpected finding to make it clearer.  **Discussion, line 333-337, page 25**  “This unexpected finding is in contrast with the literature where it has been reported that combination of antihypertensive drugs from different classes have additive or synergistic effects when used together, whereby the reduction in BP is greater than if patients were treated with either drug class alone [40].” |
| **DISCUSSION** | |
| 1. The statement that the study reported "on the quality of indicated care processes received by hypertensive patients" poses problem. The quality of counseling and other procedures have been not assessed, but only their descriptive characteristics. | We agree with the reviewer that the quality of counseling cannot be properly evaluated. In this study, the process of care covers a wider perspective than just counseling alone.  We have rephrased the statement in the discussion section to avoid misunderstanding.  **Discussion, line 231-232, page 21**  “To the best of our knowledge, our study is the first to report on documented process of care for hypertension involving multiple centres in Malaysia using patient medical records.”  We have also added a study limitation in discussion section **(Line 351-358, page 26)** to address that we are unable to measure the effectiveness of counselling in this study. The change was discussed above in our response to comment #1 in Methods. |
| 2. "The possible factors contributing to suboptimal control of hypertension could be due to patient-related factors including patients’ lifestyle and other risk factors, difficulties in adherence to prescribed regimens, limited access to care and lack of knowledge about seriousness of hypertension". This is true and significantly limits the interpretation of the presented findings. Additional information on these features should be provided. The use of intention to treat approach could also enhance the strength of the data and should be considered by the authors, or at least information on drop outs should be provided. | Yes, we agree that a more in-depth study on patient factors is warranted. In this study, we intend to look at process of care and whether they contributed to blood pressure control. It would be interesting to study the non-compliance, as in dropout, but this would not be possible with cross sectional data.  We have added a sentence on the need of further study in these areas and rephrased a sentence in the discussion section to add further clarity. It now reads:  **Discussion, line 265-268, page 22**  “Although the Chronic Care Model [31] has provided the structure needed for managing chronic disease such as hypertension, the effective implementation still requires optimal coordination, consultation, counselling and good communication skills. However, these need to be evaluated in future studies.” |
| 3. Although interesting as a text, most part of the Discussion seems to be highly speculative (please refer to lines 242 to 260). | Yes, and thank you for the comment. We attempted to speculate what could possibly explain our data. In the sense, we attempted to identify area of focus for future study.  We have removed some sentences to address your concern. It now reads:  **Discussion, line 269-283, page 22-23**  “With regards to suboptimal process of care based on the CPG [18], the fact that almost all hypertensive patients did not receive complete CV risk assessment is of great concern. The incomplete or inadequate assessment of CV risk factors may result in missed opportunities for early intervention and pose a negative impact on the morbidity and mortality of the patients. A decrease in these CV risk factors has been shown to reduce CV morbidity and mortality in both individuals with or without established CV disease [32]. Furthermore, the data from the National Health and Morbidity Surveys of Malaysia in 2015 [7] indicated that the prevalence of CV risk factors including hypertension, dyslipidaemia, T2DM, overweight or obesity and smoking is high and has been on an increasing trend. The possible causes for the poor rate of compliance to guidelines or the practice gap for the CV risk assessment were reported to be the absolute lack of physician time [33], knowledge-related factors such as lack of awareness, clinical experience and familiarity or attitude-related factors including lack of agreement, lack of outcome expectancy, self-efficacy, and motivation [29]. Nevertheless, important CV risk such as documentation of investigation on fasting blood glucose, cholesterol profile, renal function, BP status in this study were well documented.” |
| 4. The authors seemed to assume the physical activity counseling has been effectively resulted in actual practice - any information on this? | We thank the reviewer for pointing this out. Our initial phrase on “BP control” of the exercise/physical activity counseling might have caused some confusion. What we meant was those with exercise/physical activity counseling recorded have higher odds of BP control compared to those without record.  We have rephrased the discussion section (**Discussion, line 312-317, page 24)** to improve clarity. This was addressed in comment #2 for Results. |

**Responses to comments from Reviewer #2**

| **Comments** | **Author’s Response** |
| --- | --- |
| **INTRODUCTION** | |
| 1. Add an excerpt contextualizing what primary care is, for a better understanding of the work even by people who do not have knowledge in this area specifically, something important in the case of work with great informative relevance. | Thank you for the suggestion.  We have added a paragraph on primary care in the introduction section:  **Introduction, line 71-77, page 5**  “A large majority of hypertensive patients receive their care in primary care setting in Malaysia [12]. A strong coordinating role of primary care is essential to ensure adequate coordination of patient care between primary care providers, as well as coordination of patient care between primary care and other levels of health care to cope with the demand for long term care arrangements. This is of critical importance as evidence has shown that patients with chronic conditions including hypertension are better managed in primary care and this could reduce avoidable hospitalization rates [13].” |
| 2. Define a more specific objective of the study, and add the authors' hypotheses, what did you expect as a result before producing this study? | Thank you for the comment.  We have added the hypothesis as suggested and made our objectives clearer now in the introduction section.  **Introduction, line 85-87, page 5**  “We hypothesized that hypertensive patients who received care for hypertension were associated with better BP control. We also determined the extent of adherence to national clinical practice guidelines (CPG) for hypertension.” |
| **METHODS** | |
| 1. If possible, the authors will have access to this information, cite the methods of analysis and equipment that were used, in the analysis of the lipid profile, for example. | We thank the reviewer for pointing this out. However, we are unable to provide details on the methods of analysis and equipment that were used. We assumed that all the clinical parameters are measured using calibrated equipment and analyses carried out in the laboratory facilities as these 20 clinics have standard operating procedures to abide that was set by the Ministry of Health (MOH) Malaysia. |
| **RESULTS** | |
| 1. Changing the order of the tables, I believe that this would facilitate understanding, as an example:  • Table 1: Patient demographic data  • Table 2: Process of care for hypertensive patients  • Table 3: Blood pressure control status in different subgroups of hypertensive patients  • Table 4: Prescribing pattern of antihypertensive agents | We agree with the reviewer and have changed the order of the tables as suggested. |
| 2. Add the data from the excerpt “Unfortunately, less than one percent of the records documented assessment of cardiovascular risk” (page 10, line 164), in the body of the text, but not in the table. | The data on documentation of cardiovascular risk assessment was tabulated in Table 2 under the row heading “CV risk assessment in the past 1 year”.  Kindly refer to Table 2:  **Results, line 189-190, page 13-14** |
| 3. How was the definition of appropriate choice or not of antihypertensive agents? It is an important aspect that the text does not make clear. | Thank you for pointing this out. We have defined the "Inappropriate choice of antihypertensive agents" in the method to improve clarity.  **Method, line 142-150, page 8**  “Those antihypertensive drugs that were prescribed not according to criteria set in the Malaysian CPG on Hypertension 2018 were considered as inappropriate choice of antihypertensive agents [18]. These drugs were considered inappropriate choice if any of the following was fulfilled: i) alpha-blocker was prescribed as a single agent to hypertensive patients with T2DM or elderly patients ≥ 65 years old ii) beta-blocker was prescribed as a single agent to hypertensive patients with T2DM or dyslipidemia, iii) diuretic was prescribed as a single agent for hypertensive patients with T2DM or dyslipidemia, iv) angiotensin converting enzyme inhibitor (ACEI) or angiotensin receptor blocker (ARB) was not prescribed to T2DM patients with proteinuria.” |
| 4. Were patients who were undergoing lifestyle changes likewise prescribed hypertensive agents? Explain more about it in the text, it doesn't seem to be clear. | Thank you for the suggestion. We have defined hypertensive patient in lifestyle modification group and incorporated this definition (line 132-133 ) in the method section:  **Method, line 132-133, page 8**  “Those without anti-hypertensive agents prescribed but with a diagnosis of hypertension were considered to be on lifestyle modification.”  We have added information about BP status of these two groups (HPT with antihypertensive agents and HPT on lifestyle modification) in the result section, both in text and in Table 3, to further improve clarity:  **Result, line 196-197, page 15**  “Hypertensive patients with lifestyle modification appeared to have a better BP control rate compared to those on antihypertensive agents.”  As for Table 3, we have inserted the two parameters (HPT with antihypertensive agents and HPT on lifestyle modification) with results.  We also changed one of the column headers in Table 3 [from Patients achieving target blood pressure control to n(%)] due to overlook from our side. Kindly refer to Table 3 at:  **Result, line 199-200, page 16-17** |
| 5. Thinking about improving the understanding of the characteristics of the patients analyzed in this research, it would be interesting to include a table with blood pressure data, in addition to only “controlled” and “uncontrolled”. For example:   - Patients Age> 80 years (n = 517)   - X patients BP systolic 140-150 mmHg and BP diastolic 80-89.   - Y patients BP systolic 150-160 mmHg and BP diastolic 90-99. | Yes, we agree with the reviewer that tabulating BP control to various levels would be interesting. This will add further information to the pattern of BP. Nevertheless, for practical purpose, treating towards BP target is of our interest. Thus, we wish to analyse the BP control according to our objectives. |
| **DISCUSSION** | |
| 1. In the excerpt “Surprisingly, in this study, in the association between counseling on salt intake and good BP control was observed” (page 20, line 298), was this response expected? What are the possible reasons for this response? | We were surprised as this finding implies that counseling on salt intake may not be received by the patients effectively.  We have included this possible implication in the discussion section:  **Discussion, line 324-326, page 25**  “These findings suggest that counseling on salt intake, although documented, may not be received by patients effectively” |
| 2. According to the results, the authors indicate that patients who had counseling on physical exercise are more likely to have greater control of blood pressure, how was this counseling performed? | We apologized for the confusion of our statement. We have rephrased the discussion section (line 312-317) to add further clarity. It now reads:  **Discussion, line 312-317, page 24**  “Our findings indicate that hypertensive patients who had documented counseling on exercise/physical activity had 1.3 odds of having their BP controlled compared to those not receiving counseling. However, the information on how counseling was performed was not available in the medical record. We assume that the counseling was done as the usual practice in the clinic. The association of exercise/physical activity with lower BP was also reported in a previous study [37].” |
| 3. Add possible study limitations. | We have added a few points on limitations and rephrased the discussion section. It now reads:  **Discussion, line 346-360, page 26**  “There were some limitations in this study. First, the cross-sectional study design cannot be used to establish causal and effect relationship between the factors and outcome. Second, since it is a record-based study, we did not have some of the patients’ information including the level of education, occupation or socioeconomic status which may influence patients’ self-management such as medication adherence or compliance to lifestyle intervention to a certain extent. Third, the information on counseling was limited to types of counseling documented in the medical records. It would be difficult to capture the effectiveness of counseling given to the patients during the doctor-patient consultation session. Thus, we assumed that counseling was delivered accordingly by the attending primary care doctors. Similarly, information on the dose and duration of the antihypertensive agents were not extracted because it was assumed that the dose titration process will occur over time, aiming at BP control. Hence, it is not feasible to measure antihypertensive medication titration on BP control with our cross-sectional study design. Fourth, the assumption of the process of care and prescribing practices not documented in the medical records are considered as not done and may underestimate the actual quality of care received by the patients.” |

**Responses to comments from editor (Dr Anderson Saranz Zago)**

| 1. Please ensure that your manuscript meets PLOS ONE's style requirements, including those for file naming. | Thank you. We have made the necessary changes. |
| --- | --- |
| 2. Please state the date upon which the data used in this survey was accessed. In addition, please refer to any post-hoc corrections to correct for multiple comparisons during your statistical analyses. If these were not performed please justify the reasons. Please refer to our statistical reporting guidelines for assistance | Thank you for your comment. The finalized dataset was used for the present study after data cleaning was performed for EnPHC-EVA: Facility study. It was accessed in September 2019.  In this study, we did not perform multiple comparisons, hence we did not perform any post-hoc corrections. |
| 3. We note that you have indicated that data from this study are available upon request. PLOS only allows data to be available upon request if there are legal or ethical restrictions on sharing data publicly. | We understand the concern and rephrased the statement to express our limitation:  “All relevant data are provided within the manuscript. Individual level data cannot be made publicly available due to ethical and patient confidentiality restrictions. The data collected within this study is managed in accordance with the Personal Data Protection Act in Malaysia and is subject to ethical restrictions under the Medical Research and Ethics Committee, Ministry of Health Malaysia. Data is available on request and interested researchers may address data access requests to the Institute for Clinical Research at [contact@crc.gov.my](mailto:contact@crc.gov.my).” |
