# Supplementary material for: Quality of hypertension management in public primary care clinics in Malaysia: An update
Source: PLoS One. 2020 Aug 11;15(8):e0237083. doi: 10.1371/journal.pone.0237083 (PMC7418969; doi:10.1371/journal.pone.0237083)
Supplement: Attachment — Submitted filename: review on PLOS ONE manuscript.docx [file pone.0237083.s003.docx]

The present study aimed to assess management, and the association between the care process and the blood pressure control of hypertensive patients in primary care clinics in Malaysia.

**INTRODUCTION**

Previously, the authors were asked to add a passage in the introduction talking about “Primary Care”, and to define objectives more clearly, in order to avoid misinterpretation. In addition, the authors agreed in the excerpts:

Introduction, line 71-77, page 5:

“A large majority of hypertensive patients receive their care in primary care setting in Malaysia [12]. A strong coordinating role of primary care is essential to ensure adequate coordination of patient care between primary care providers, as well as coordination of patient care between primary care and other levels of health care to cope with the demand for long term care arrangements. This is of critical importance as evidence has shown that patients with chronic conditions including hypertension are better managed in primary care and this could reduce avoidable hospitalization rates [13].”

Introduction, line 85-87, page 5:

“We hypothesized that hypertensive patients who received care for hypertension were associated with better BP control. We also determined the extent of adherence to national clinical practice guidelines (CPG) for hypertension.”

**METHODS**

Regarding the methods, the authors were asked to provide information about the analyzes performed. This was not possible, but they justified and guaranteed the standardization of the analysis procedures of the clinics.

**RESULTS**

Regarding the results, it was suggested to the authors that they change the order of the tables in this section in order to facilitate the understanding of the study, and this was done. In addition, to specify how was the definition of adequate choice or not of antihypertensive agents. The authors included or excerpt:

Method, line 142-150, page 8:

“Those antihypertensive drugs that were prescribed not according to criteria set in the Malaysian CPG on Hypertension 2018 were considered as inappropriate choice of antihypertensive agents [18]. These drugs were considered inappropriate choice if any of the following was fulfilled: i) alpha-blocker was prescribed as a single agent to hypertensive patients with T2DM or elderly patients ≥ 65 years old ii) betablocker was prescribed as a single agent to hypertensive patients with T2DM or dyslipidemia, iii) diuretic was prescribed as a single agent for hypertensive patients with T2DM or dyslipidemia, iv) angiotensin converting enzyme inhibitor (ACEI) or angiotensin receptor.”

Previously, in relation to patients who have undergone changes in lifestyle, they were asked to detail these advice, as it would not have been clear. In addition, the authors included the excerpts:

Method, line 132-133, page 8:

“Those without anti-hypertensive agents prescribed but with a diagnosis of hypertension were considered to be on lifestyle modification.”

Result, line 196-197, page 15:

“Hypertensive patients with lifestyle modification appeared to have a better BP control rate compared to those on antihypertensive agents.”

**DISCUSSION**

As for the discussion, it was suggested to the authors to add the study's hypothesis regarding the advice on salt intake and the good control of arterial hypertension. In addition, it was suggested to detail how the physical activity counseling was carried out in the study, as it would not have been clear. And the authors agreed and included the following excerpts:

Discussion, line 324-326, page 25:

“These findings suggest that counseling on salt intake, although documented, may not be received by patients effectively.”

Discussion, line 312-317, page 24:

“Our findings indicate that hypertensive patients who had documented counseling on exercise/physical activity had 1.3 odds of having their BP controlled compared to those not receiving counseling. However, the information on how counseling was performed was not available in the medical record. We assume that the counseling was done as the usual practice in the clinic. The association of exercise/physical activity with lower BP was also reported in a previous study [37].”
