# Supplementary material for: Quality of hypertension management in public primary care clinics in Malaysia: An update
Source: PLoS One. 2020 Aug 11;15(8):e0237083. doi: 10.1371/journal.pone.0237083 (PMC7418969; doi:10.1371/journal.pone.0237083)
Supplement: Attachment — Submitted filename: Response to Reviewer.docx [file pone.0237083.s004.docx]

Dear Editor and Reviewer #1,

The current study aimed to assess the process of care for hypertension management and the association between process of care and blood pressure control in primary care clinics in Malaysia.

**INTRODUCTION**

The authors were asked to rephrase the hypothesis in lines 85-86: “We hypothesized that hypertensive patients who received care for hypertension were associated with better BP control”. The reviewer felt that it did not reflect the main purpose or results of the study. The authors agree with the reviewer and have omitted the hypothetical statement. It now reads:

Introduction, Line 78-86, page 5:

“In Malaysia, improvement in process of care has been undertaken over the years with better establishment of non-communicable disease care such chronic care concepts, diabetic registry, nurse educator and better range of antihypertensive medication. Although there were some evaluations on hypertension, process of care evaluation was last done 10 years ago [11]. Most of the evaluations on hypertensive care focused on the burden of hypertension in the Malaysian population [14–16]. Similarly, evaluations on process of care and BP control in low- and middle-income countries are scarce. Therefore, we aimed to evaluate the current process of care for hypertension management as well as to explore the association between process of care and BP control among hypertensive patients in public primary care clinics in Malaysia.”
